# Fluid structure interaction study of non-Newtonian Casson fluid in a bifurcated channel having stenosis with elastic walls

**DOI:** 10.1038/s41598-022-16213-3

**Published:** 2022-07-18

**Authors:** Hasan Shahzad, Xinhua Wang, Abuzar Ghaffari, Kaleem Iqbal, Muhammad Bilal Hafeez, Marek Krawczuk, Wiktoria Wojnicz

**Affiliations:** 1 Faculty of Materials and Manufacturing, College of Mechanical Engineering and Applied Electronics Technology, Beijing University of Technology, Beijing, China; 2 Division of Science and Technology, Department of Mathematics, University of Education, Lahore, 54770 Pakistan; 3 Department of Mathematics, CEMAT Instituto Superior Tecnico Ulisboa, Av. Rovisco Pais 1, 1049-001 Lisboa, Portugal; 4 Faculty of Mechanical Engineering and Ship Technology, Institute of Mechanics and Machine Design, Gdansk University of Technology, Narutowicza 11/12, 80-233 Gdańsk, Poland

**Keywords:** Applied mathematics, Computational models

## Abstract

Fluid–structure interaction (FSI) gained a huge attention of scientists and researchers due to its applications in biomedical and mechanical engineering. One of the most important applications of FSI is to study the elastic wall behavior of stenotic arteries. Blood is the suspension of various cells characterized by shear thinning, yield stress, and viscoelastic qualities that can be assessed by using non-Newtonian models. In this study we explored non-Newtonian, incompressible Casson fluid flow in a bifurcated artery with a stenosis. The two-dimensional Casson model is used to study the hemodynamics of the flow. The walls of the artery are supposed to be elastic and the stenosis region is constructed in both walls. Suitable scales are used to transform the nonlinear differential equations into a dimensionless form. The problem is formulated and discretized using Arbitrary Lagrangian–Eulerian (ALE) approach. The finite element method (FEM) technique is used to solve the system of equations, together with appropriate boundary conditions. The analysis is carried out for the Bingham number, Hartmann number, and Reynolds number. The graphical results of pressure field, velocity profile, and load on the walls are assessed and used to study the influence of hemodynamic effects on stenotic arteries, bifurcation region, and elastic walls. This study shows that there is an increase in wall shear stresses (WSS) with increasing values of Bingham number and Hartmann number. Also, for different values of the Bingham number, the load on the upper wall is computed against the Hartmann number. The result indicate that load at the walls increases as the values of Bingham number and Hartmann number increase.

## Introduction

Featuring the human circulatory system, the bio-fluid dynamic has grown rapidly in recent decades, particularly concerning atherosclerosis diagnosis and etiology. Due to a buildup of fatty deposits like calcium, arterial stenosis occurs. Atherosclerotic plaque is determined by the geometry of the arteries. The most common places for stenosis to develop are the curvatures, crossings, and forks of the medium and large arteries. The study of atherosclerosis and the patterns of blood flow in stenotic or bifurcated arteries has witnessed a substantial increase in interest in recent years. Blood composition, cell concentrations, and artery geometry all have a direct impact on the arterial system's flow characteristics. Several research has sought to better predict a flow of blood in a bifurcated artery by implementing Newtonian and non-Newtonian fluids. According to vein geometries and blood rheological behavior in the circulation, theoretical and empirical research of blood flow through arterioles are complex task with numerous challenges^1^. Blood flow's is complex rheological dynamics that cannot be predicted by any structural mode. Therefore to analyze blood hemodynamics, researchers formulate new methods^2^. Blood is a mixture of cells suspended in a fluid^3,4^. Red blood cells round the greater volume of blood^5^. According to Hunter^6^, the blood was assumed to be incompressible, homogeneous, and Newtonian fluid. Due to the elasticity of the vessel wall, blood flow was unsteady in this model. A finite difference method was used to calculate the blood flow. Also, a one-dimensional heat equation was used. Computed results were estimated by assuming that there was no heat loss around the artery.

There are many non-Newtonian fluid models, but one of the most well-known is the Casson Fluid (CF) model^7^. Shear stress and strain are nonlinearly related in the Casson model. This model is used to study the blood flow, paints industry, manufacturing of medicine, and synthetic lubricants. Misra and Pandey^8^ used CF model to study the peristaltic blood transport in small vessels. The CF model is also used to describe the core region of the blood flow in mathematical model of small vessels in their mathematical model. It is worth noting that human blood flow is essentially pulsed and irregular due to its cyclic nature, and this poses a unique challenge to both computational and experimental science. To date this CF model is the best formulation for predicting a flow of blood^9–11^. Khair et al.^12^ studied the pulsatile blood flow in a constricted channel. Chakravarty and Mandal^13^ studied the blood flow in a stenosis of tapered artery. For further study on CF model and its application the readers are referred to^14–16^ and references therein.

Magnetohydrodynamics (MHD) is a part of fluid dynamics that does incorporate the fluid’s magnetization or polarization while studying fluid dynamics in the magnetic field. MHD has a wide range of potential applications in bioengineering and medicine^17^. Sharma et al.^18^ reported that MHD can be controlling parameter for blood velocity. Considering the micropolar fluid non-linear model^19^, Shit and Roy found that enhancing the effects of induced magnetic field on blood flow via a confined channel the blood flow velocity at the centerline is reduced and raised the pressure gradient. Diviya et al.^20^ studied the hemodynamics of the MHD peristaltic process of non-Newtonian fluid with mass and heat transfer. Their studies show that an increase in variable viscosity parameters accelerates the flow hence bolus size increases. Pulsatile flow analysis has gained a lot of attention because of its applications in respiratory system, circulatory systems, microelectromechanical system, reciprocal pumps, vascular diseases, and internal combustion engines^21–25^. Malathy and Srinivas^26^ used perturbation method to investigate the MHD pulsating flow between two permeable beds. A perturbation technique was used by Srinivas et al.^27^ to study the non-Newtonian pulsative flow in a porous channel. Recent studies by Bilgi and Atalik^28^ have explored the elastic properties of blood for pulsative arterial hemodynamics and risk indicators for aneurysm rift to their impact on velocity, stress fields, and vorticity.

Many physiological processes in the body are influenced by the FSI. For example, pulmonary airway reopening and closure, flow-rate limitation and wheezing during forced expiration, snoring and phonation, pulse wave propagation in arteries, flow-induced deformation, and ultimate rupture of arterial cerebral aneurysms, etc. To represent the FSI in the pulmonary arteries, Liu et al.^29^ proposed employing a unified continuum and an interdisciplinary variant formula. A quasi-direct approach was used to solve the FSI problem to assess velocity and pressure. To compute the solid displacement and mesh motion a segregated algorithm is used. Using biological approaches, Foong et al.^30^ studied the numerical similarity of blood flow within the artery under continuous heat flux. The study shows that non-Newtonian blood flow changes into Newtonian blood flow properties by replenishing fluid and electrolytes in the bodily arteries, which promotes the heat transfer in blood flow and causes blood flow temperature to be reduced. Heat transmission through oscillatory blood circulation in an incised permeable artery was studied by Ogulu and Abbey^31^. Khaled and Vafaei^32^ studied the concept of transport in biological tissue and proposed new model incorporating heat transfer and thermic biology equation. Recently, Shahzad et al.^33^ used power-law fluid to study the hemodynamics of blood flow in a stenotic artery with elastic walls by using ALE approach to couple equations. The results show that there is an increase in load on the walls for the shear thickening case.

Casson model widely used over a long ranges of shear rates for the study of blood rheology was not implemented in fluid solid interaction in an artery. In the presented study, a Casson model is considered to investigate the hemodynamic effects of the blood flow flowing through a bifurcated artery having elastic walls. The scope of the study involves testing impact the Bingham number, Reynolds number, and Hartmann number on the hemodynamics of the artery, stenotic region, and elastic walls. In the next section governing equations are modeled and converted into dimensionless form. In problem setup section the geometry of the problem and solution approach is explained. In the final section, a conclusion is drawn based on the results.

## Mathematical modeling

We studied the flow of two-dimensional, non-Newtonian incompressible fluid through the double stenosis bifurcated artery. The artery walls are considered to be linearly elastic. A magnetic field is applied toward the axial direction of the flow. Considering, motions of solid and fluids described by Lagrangian and Eulerian approaches, one can state that ALE is a more general method mixing fluid and solid domains (FSI). Governing equations for FSI case are written as

1 $$\nabla \cdot \widetilde{{\varvec{u}}}=0,$$

2 $${\rho }_{f}\left(\left(\widetilde{{\varvec{u}}}-{\widetilde{{\varvec{u}}}}_{{\varvec{s}}}\right) \cdot \nabla \widetilde{{\varvec{u}}}\right)=-\nabla \widetilde{p}+\nabla \cdot {\varvec{\tau}}+\overline{{\varvec{f}} },$$

where ${\overline{{\varvec{f}} }=[\sigma }^{*}{\widetilde{u}}_{x}{\widetilde{B}}^{2},0]$. The blood may be modeled using the original Casson fluid constitutive equation over a diverse range of shear rates^34^

3 $$\sqrt{{\varvec{\tau}}}=\sqrt{{\tau }_{y}}+\sqrt{{\mu }_{p}{\varvec{\gamma}},}$$

where , ${\tau }_{y}$, ${\mu }_{p}$, and ${\varvec{\gamma}}$ represents the yield stress, Casson viscosity, and shear strain rate respectively. The shear strain rate is defined as

4 $${\varvec{\gamma}}=\nabla {\varvec{u}}+{\left(\nabla {\varvec{u}}\right)}^{T},$$

The discontinuous nature of the Casson model makes it challenging to implement in numerical simulation. Using a strategy that was previously utilized to overcome a Bingham plastic fluid singularity^35^, an improved continuous version^36^ can be used

5 $${\varvec{\tau}}={\left[\sqrt{{\mu }_{\infty }}+\sqrt{\frac{{\tau }_{y}}{\left|{\varvec{\gamma}}\right|}}\left(1-{e}^{-\sqrt{m \left|\gamma \right|}}\right)\right]}^{2}{\varvec{\gamma}},$$

where $m$ is model constant and ${\mu }_{\infty }$ is asymptotic apparent viscosity. In^37^ was established that when $m>100,$ the Eq. (6) is a good approximation of the Casson model. The apparent viscosity of the Casson model can be defined as

6 $$\overline{\eta }=\frac{{\varvec{\tau}}}{{\varvec{\gamma}}}={\left[\sqrt{{\mu }_{\infty }}+\sqrt{\frac{{\tau }_{y}}{\left|{\varvec{\gamma}}\right|}}\left(1-{e}^{-\sqrt{m \left|\gamma \right|}}\right)\right]}^{2}$$

Equations for the elastic structure domain are

7 $$\nabla \widetilde{{\varvec{\sigma}}}=0,$$

As elastic walls are exposed to a stress tensor (caused by fluid pressure) the walls are deformed

8 $$\widetilde{{\varvec{\sigma}}}={J}^{-1}{\varvec{F}}{\varvec{S}}{{\varvec{F}}}^{T}$$

where ${\varvec{F}}=\left(1+\nabla {\widetilde{d}}_{s}\right), J=det \cdot \left({\varvec{F}}\right)$, ${\varvec{S}}$ is the 1st Piola–Kirchhoff stress tensor which is related to strain $\varepsilon$ as follows

9 $$S=C:\left(\varepsilon \right),\quad \varepsilon =\frac{1}{2}\left(\nabla {\widetilde{d}}_{s}+\nabla {\widetilde{d}}_{s}^{T}+\nabla {\widetilde{d}}_{s}^{T}\nabla {\widetilde{d}}_{s}\right).$$

where $$C=C\left(E,\nu \right)$$.

$C$ is elasticity tensor and “:” is the double-dot tensor product. The boundary conditions for FSI at the wall surface are continuity of dynamic movement and kinematic forces.

To make the analysis more general, the governing equations are converted into dimensionless form by non-dimensional variables ${\varvec{u}}$**,** $p$, and ${\varvec{\tau}}$ and choosing $h$ and $U$ as reference length and reference velocity respectively. Because of the above discussion Eqs. (1–8) takes the form

10 $$\nabla \cdot {\varvec{u}}=0,$$

11 $$Re \left({\varvec{u}}-{{\varvec{u}}}_{{\varvec{s}}}\right) \cdot \nabla {\varvec{u}}=-Re\boldsymbol{ }\nabla p+\nabla \cdot {\varvec{\tau}}+{\varvec{f}},$$

where $${\varvec{f}}=[-{Ha}^{2} u,0]$$ and

12 $${\varvec{\tau}}={\left[1+\sqrt{\frac{Bn}{\left|{\varvec{\gamma}}\right|}}\left(1-{e}^{-\sqrt{M \left|\gamma \right|}}\right)\right]}^{2}{\varvec{\gamma}},$$

where $Re=\frac{{\rho }_{f}hU}{{\mu }_{\infty }}$ is Reynolds number, $Ha=\frac{{\sigma }^{*}{h}^{2}{\widetilde{B}}^{2}}{{\mu }_{\infty }}$ is Hartmann number, $Bn=\frac{{\tau }_{y}h}{{\mu }_{\infty }U}$ is Bingham number and $m$ in dimensionless form is defined as $M=\frac{mU}{h}$. The viscosity in dimensionless form is defined as

13 $$\eta ={\left[1+\sqrt{\frac{Bn}{\left|{\varvec{\gamma}}\right|}}\left(1-{e}^{-\sqrt{M \left|\gamma \right|}}\right)\right]}^{2},$$

The dimensionless equation for elastic structure domain is defined as

14 $$\nabla{\varvec{\sigma}}=0.$$

A parabolic inflow velocity with ${u}_{max}=0.6$ is considered at the inlet and at outlets $p=0$ boundary condition is imposed.

## Problem setup

### Flow configuration

**Figure 1:**
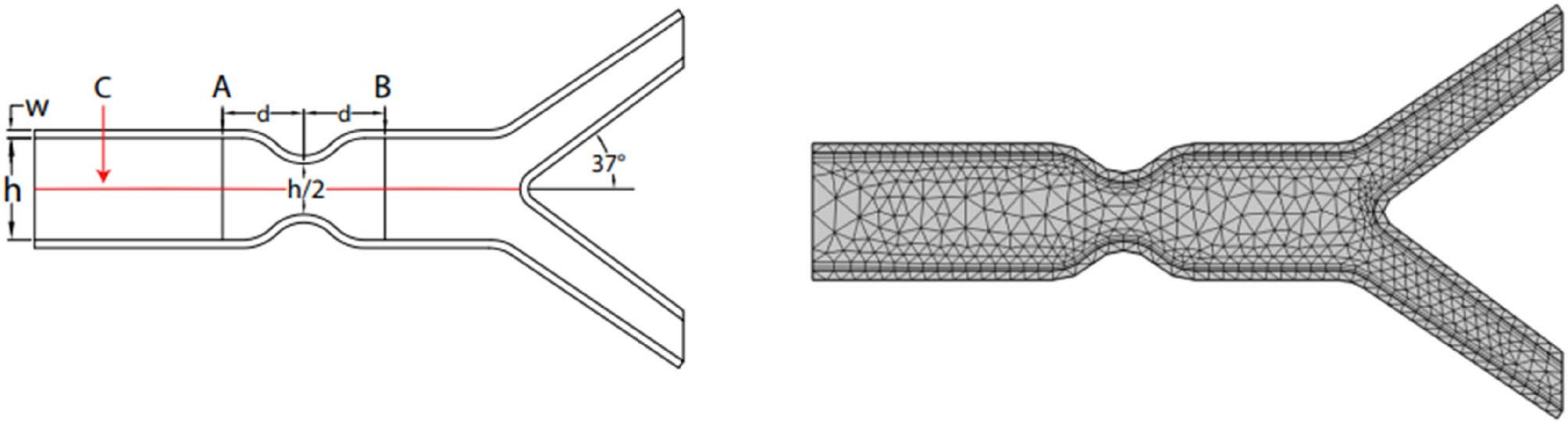
Problem formulation.

In Fig. 1, a prototype geometric model is considered. The computational domain includes a symmetrical bifurcation and stenosis. It is assumed that walls are made of isotropic and linear elastic materials characterized by the Poisson ratio $\nu$ and Young’s modulus E. The relationship between Young’s modulus and the Poisson ratio is defined as

15 $${\lambda }_{l}=\frac{E \nu }{\left(1+\mu \right)\left(1-2\nu \right)},\quad \nu =\frac{{\lambda }_{l}}{2\left(\mu +{\lambda }_{l}\right)}.$$

where $\nu , E, \mu ,$ and ${\lambda }_{l}$ are Poisson ratio, Young’s modulus, shear modulus and Lamé coefficient respectively. Where $\nu =0.49$, and $E=5\times {10}^{5}$^33^.

The inner diameter of the artery $h$ is shown in Fig. 1. It was stated that this diameter equaled 1 cm and it shrinks to 50% in the stenosis zone. The width of the elastic wall is constant and equals $w=0.1cm$ and the bifurcation artery is inclined at the 37 °C is the central longitudinal axis along which the pressure is tested. The points A and B are chosen only to predict a behavior of the velocity profile before and after stenosis respectively.

### Solution methodology

The Eqs. (10–14) are nonlinear and canot be solved in analytical way. In this study the ALE method is used to solve the above problem. This approach combines the Lagrangian method’s facility of moving boundary domain with the Eulerian method’s facility of holding a fixed domain. Donea and Giuliani^38^, Donea and Huerta^39^, Kuhl et al.^40^, and Mazumder^41^ provide more information on the ALE approach implementation. The accuracy of the solution can be improved by using a hybrid mesh of triangular and rectangular components. A Galerkin finite element technique is used to convert the FSI problem into a weak form and discretized. The element pair P_2_-P_1_ is chosen to approximate the pressure, velocity, and elastic walls behavior. The Newton’s method is used to solve the nonlinear algebraic system of equations. The nonlinear iteration convergence criteria are defined as:

$$\left|\frac{{\zeta }^{n-1}-{\zeta }^{n}}{{\zeta }^{n+1}}\right|<{10}^{-6}$$

where $\zeta$ represents the general solution component and $n$ is the number of iterations. Figure 1 (right) depicts the problem's coarser lever mesh grid. The problem domain is subdivided into a finite number of elements. P_1_ and P_2_ to approximate the domain's elements. A quadrilateral and triangular grid are used to create the mesh. To ensure that results are independent of the number of mesh elements. A grid independence study was carried out by computing WSS for the upper elastic wall. The numerical values of WSS from coarser (level 1) to extremely fine (level 6) were computed at $Re=200, Ha=0 \,\, {\text{and}}\,\, Bn=4$. The absolute error decreases when the refinement level were increased and was minimum at level 6. Therefore, all the simulations were performed at level 6. The number of elements and degree of freedom at each level are shown in Table 1.

**Table 1:** Mesh statistics for various refinement levels.

Level	# Elements	# DOF	Total WSS upper wall	Error
1	1904	10,871	0.44897	–
2	2467	13,731	0.44955	0.00058
3	3503	19,106	0.45012	0.00057
4	8941	48,458	0.45116	0.00104
5	24,889	132,054	0.45134	0.00020
6	27,495	145,581	0.45133	0.00001

### Code validation

**Figure 2:**
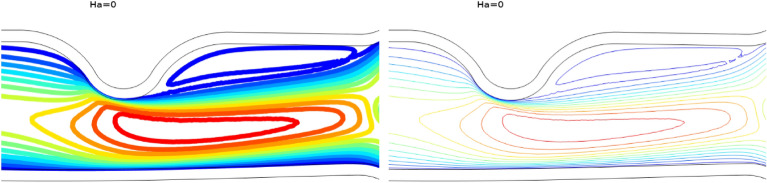
Comparison for the contour plots at $Re=300$, Anwar et al.^42^ (left) and present (right).

Once grid independence is established, the validation of code is presented against the results of Anwar et al.^42^ for contour plots and velocity magnitude and are shown in Fig. 2 and Table 2 respectively. The comparison demonstrated the accuracy of our results, and a good agreement among the respective results is obtained, which ensures that the results obtained from the present study are reliable for accuracy check.

**Table 2:** Velocity magnitude comparison.

$Ha$	$Re=300$	$Re=300$
	Fsi case (Anwar et al.^42^)	Fsi (present resutls)
0	0.5556	0.5554
8	0.5172	0.5170
10	0.4982	0.4980
12	0.4731	0.4731

## Results and discussion

To study the hemodynamics of the flow the numerical study of non-Newtonian biomagnetic blood flow flowing through a bifurcated channel is carried out by using the two-dimensional CF model. The walls of the artery are assumed to be elastic and the stenosis region is constructed in both walls. Suitable scales are used to transform the nonlinear differential equations into a dimensionless form. The problem is formulated and discretized using the ALE approach. The system of equations is solved using the FEM technique along with appropriate boundary conditions. To get a better view of the problem, the numerical solution is derived for various values of parameters involved. The ranges of the parameters used in the study are $200\le Re\le 1000$, $0\le Bn\le 4,$ and $0\le Ha\le 10$.

**Figure 3:**
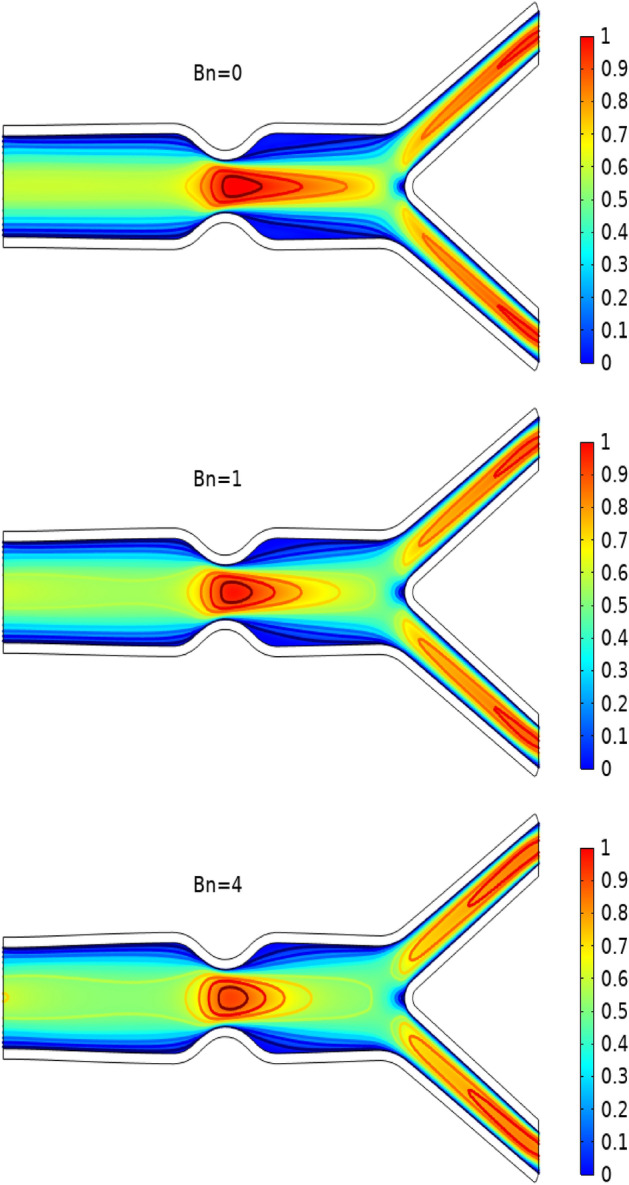
Velocity profile at Re = 200 for variation of Bn.

**Figure 4:**
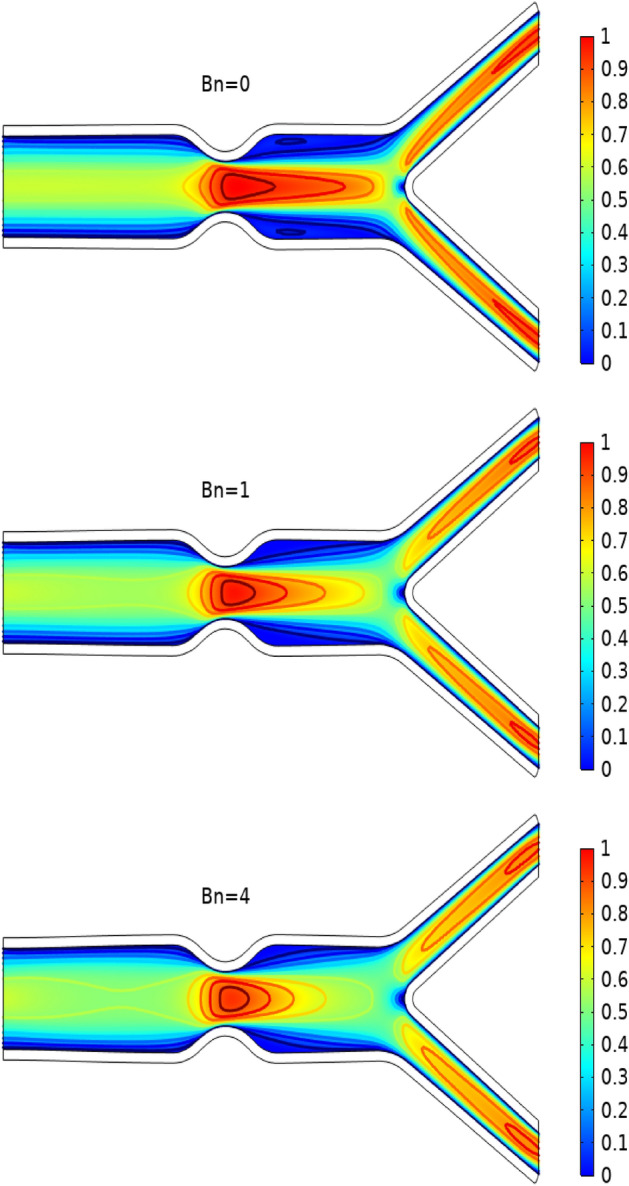
Velocity profile at Re = 400 for variation of Bn.

**Figure 5:**
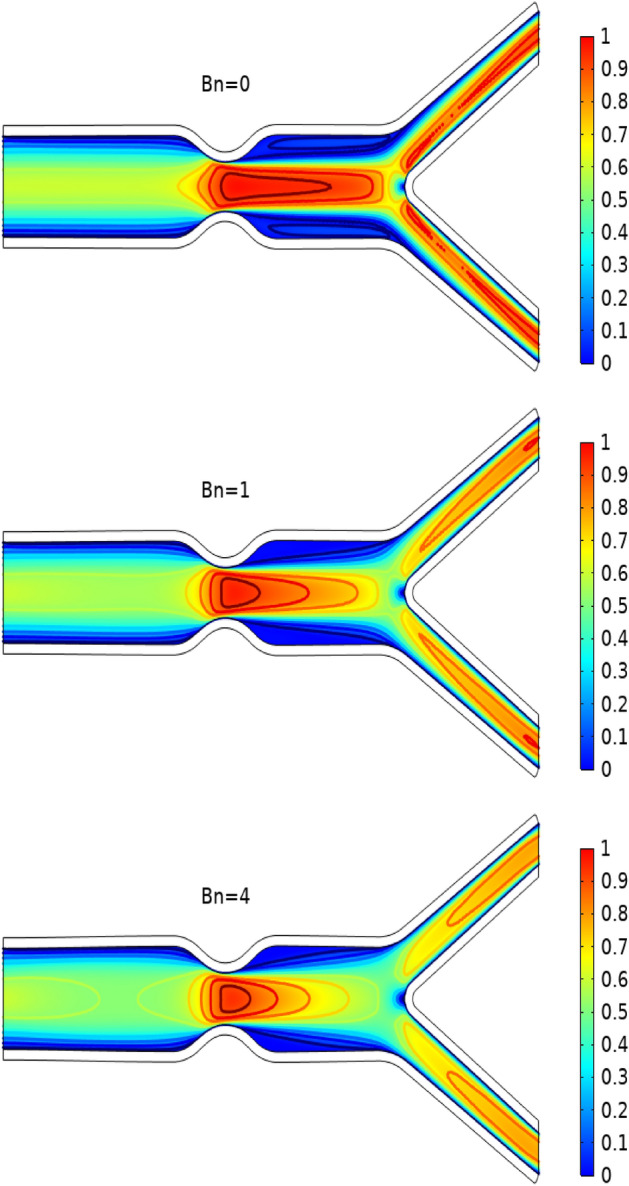
Velocity profile at Re = 1000 for variation of Bn.

**Figure 6:**
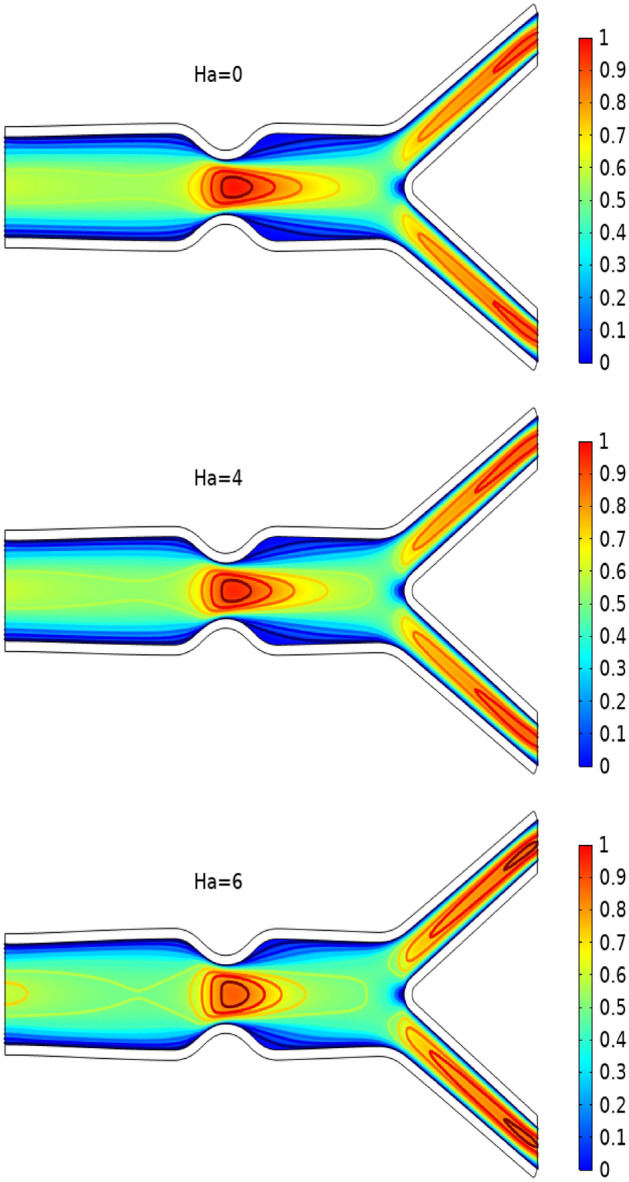
Velocity profile at Re = 200 for variation of Ha.

**Figure 7:**
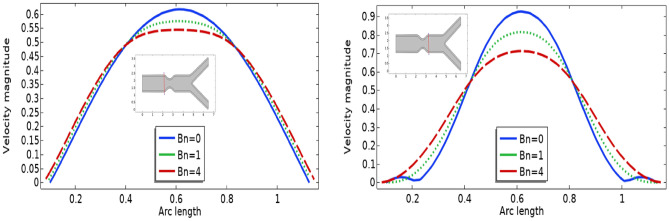
Velocity profile for different Bn.

**Figure 8:**
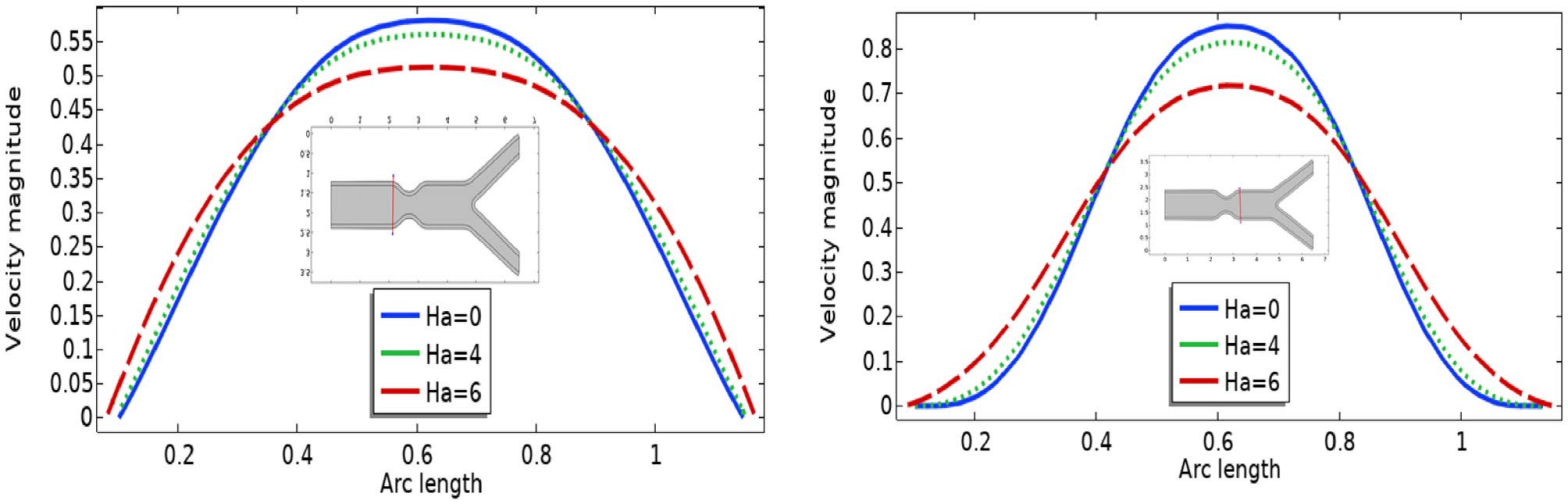
Velocity profile for different Ha.

In Figs. 3, 4 and 5 streamlines of blood flow are depicted for different values of $Bn$ at $Re=200, 400,$ and 1000 respectively. Increasing values of $Bn$, the cavity area located near the stenosis region increases due to augmented pressure at the walls. Also for higher Reynolds number the recirculation near the stenosis increases. From the physical point of view this means the viscous forces within a blood flow increase for higher Reynolds number that retards the velocity of the flow. Also, the velocity magnitude is maximum for $Bn=0$ (Newtonian case). Figure 6 plots the velocity for the variation of the Hartmann number for constant $Re=200$ and $Bn=1$. The velocity of the blood is maximum for Ha = 0. WSS increases with increasing values of Ha. A reasonable deformation can be detected for the variation of the $Ha$ due to the elastic nature of walls. In Figs. 7 and 8 velocity magnitude at locations A and B is plotted for different values of $Bn$ and $Ha$ respectively. Near the walls of the channel, velocity increases with increasing values of $Bn$. When the distance between the elastic wall and fluid flow increases the velocity starts decreasing for increasing values of $Bn$. Also, velocity magnitude is maximum for the Newtonian case ($Bn=0$). The same trend can be seen for the variation of the Hartmann number and is depicted in Fig. 8.

**Figure 9:**
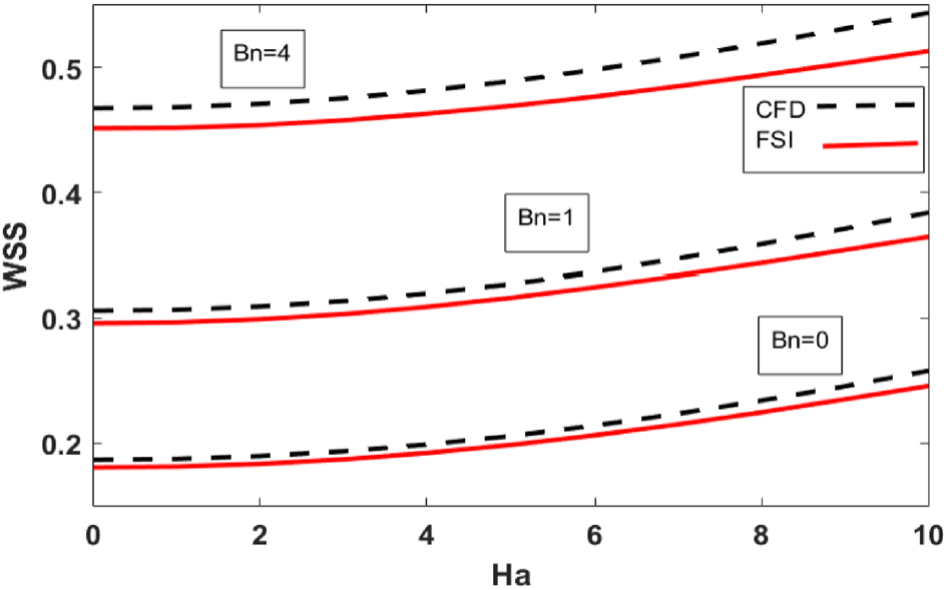
WSS along the upper wall vs Ha for different Bn.

**Figure 10:**
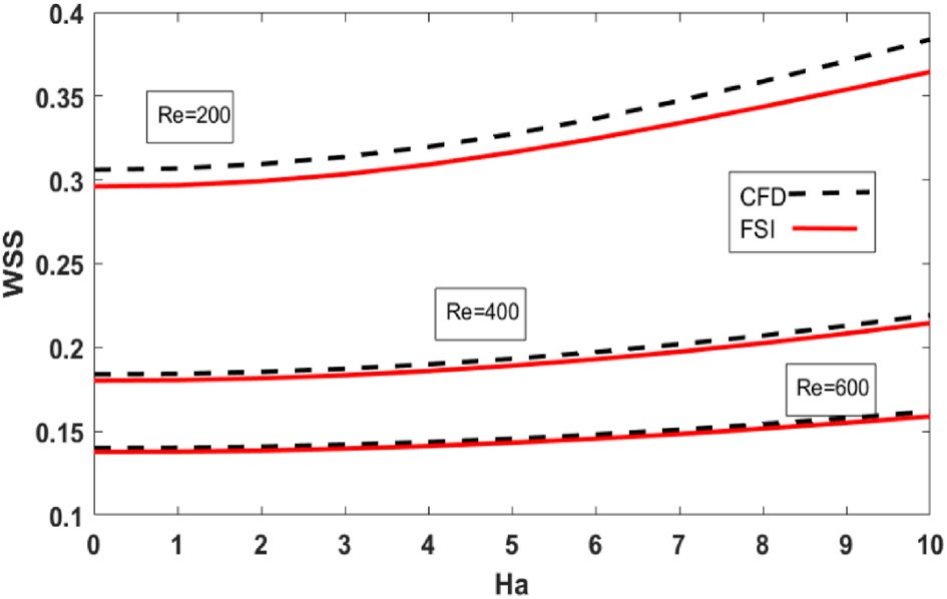
WSS along the upper wall vs Ha for different Re.

Mechanical features of the bifurcated channel are critical since they are directly related to flow pattern, recirculation area, and WSS. The distribution of WSS along the upper wall for the variation of Reynolds number and Hartmann number are shown in Table 3. Also, a comparison is made for FSI (elastic wall) and CFD (rigid wall). WSS drops in the FSI scenario compared to the CFD case where the walls of the artery are considered to be rigid. In case of increasing Reynolds number WSS decreases while Hartmann number has the opposite effect on the value of WSS. Figures 8 and 9 plots the WSS against Ha for variation of Bingham number and Reynolds number respectively. Also, a comparison is made between FSI and CFD cases. WSS is minimum for the Newtonian case ($Bn=0$). Variations in $Bn$ give rise to the WSS (see Fig. 9). In Fig. 10 the influence of the Reynolds number on WSS along the upper wall is plotted. An increase in Reynolds number increases the viscous forces inside the fluid which retards the velocity of the fluid hence WSS decrease with increasing Reynolds number.

**Table 3:** Wall shear stresses for variation of Ha and Re.

$Ha$	$Re=200$		$Re=400$		Re = 600	
	CFD case	FSI case	CFD case	FSI case	CFD case	FSI case
0	0.306026	0.296094	0.18397	0.180241	0.139965	0.137741
2	0.309471	0.299257	0.185416	0.181606	0.140804	0.138476
4	0.319744	0.309125	0.189908	0.185925	0.143505	0.141158
6	0.336633	0.324699	0.197262	0.192986	0.148063	0.145541
8	0.358729	0.343663	0.207165	0.202546	0.154248	0.151569

**Figure 11:**
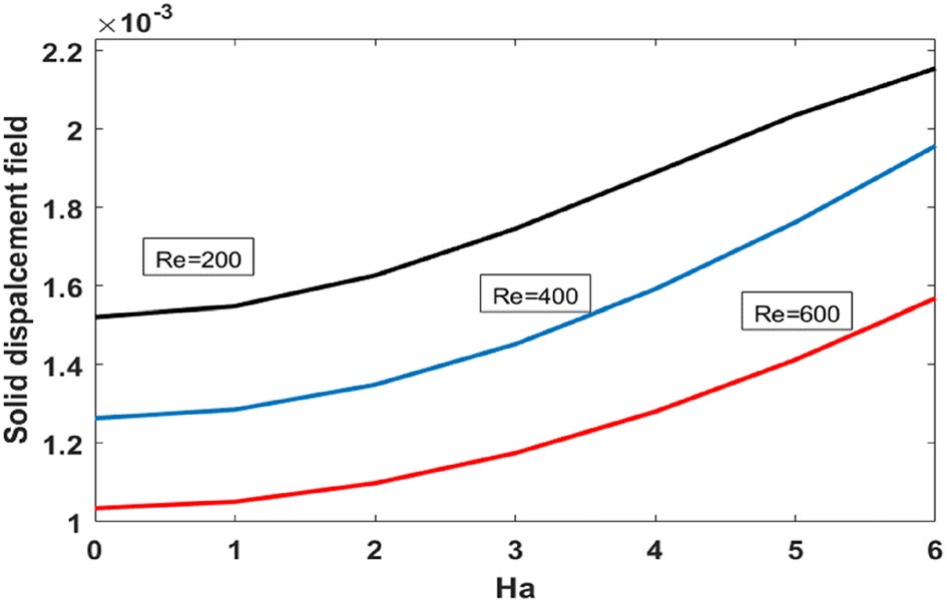
Upper wall’s displacement field vs Ha for different Re.

**Figure 12:**
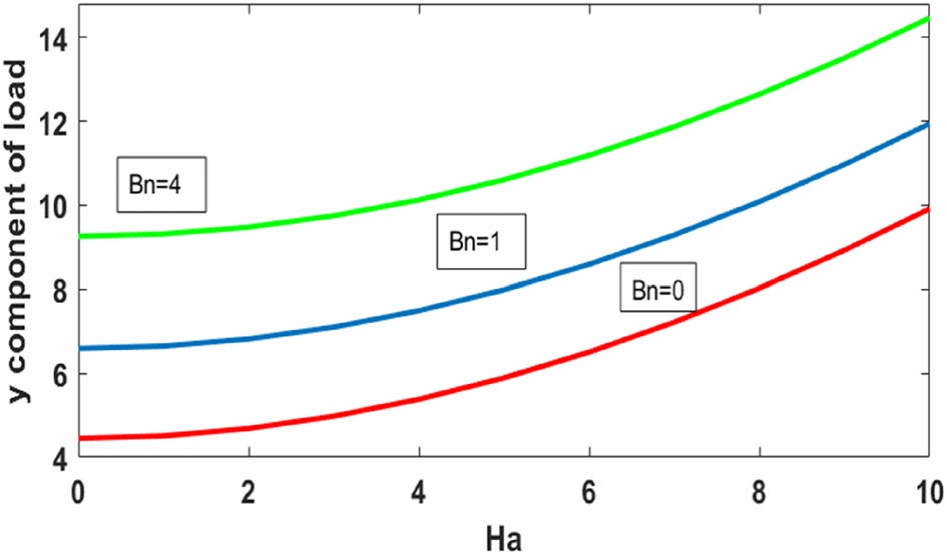
Fluid load on the upper wall vs Ha for different Bn.

Figure 11 plots the upper wall displacement field vs Hartmann number for the variation of Reynolds number. It is evident that a displacement field increases for increasing values of $Ha$. An increase in Reynolds number decreases the displacement field. The y component of load against Hartmann number for the variation of Bn is plotted in Fig. 12. We found that the y component wall load is minimum for the Newtonian case ($Bn=0$) and increases with increasing values of $Bn$.

## Conclusion

This study presents results of FSI study aimed to model interaction between double stenosis bifurcated channel and blood flow implemented as non-Newtonian incompressible fluid described by two-dimensional CF model. The channel’s walls are assumed to be elastic. The magnetic field is applied along the axial direction of the flow. The system of differential equations is transformed into a dimensionless form by utilizing suitable scales. ALE-based FEM is used to discretize the system of governing equations. The study’s primary findings are noted as follows:

- The WSS is higher for rigid wall scenarios than elastic wall cases.
- As the Reynolds number increases, the viscous forces inside the fluid increase which retards the fluid velocity inside the artery.
- The WSS decreases for increasing values of Reynolds number.
- WSS is minimum for the Newtonian case ($Bn=0$), as $Bn$ increases so does the WSS increases.
- As the values of $Bn$ and $Ha$ increase, so does the load on the wall.

## Data Availability

The datasets used and/or analysed during the current study are available from the corresponding author on reasonable request.

## List of symbols

$${\lambda }_{l}$$: Lamé coefficient
$$\mu$$: Shear modulus
$${\tau }_{y}$$: Yield stress
$$\nu$$: Poisson ratio
$${\mu }_{p}$$: Casson viscosity
${\mu }_{\infty }$: Asymptotic apparent viscosity
$$E$$: Young’s modulus
$${\rho }_{f}$$: Fluid density
$$p$$: Pressure
$${\sigma }^{*}$$: Electrical conductivity
$$\widetilde{B}$$: Magnetic field
$\widetilde{{\varvec{u}}}=[\widetilde{u},\widetilde{v}]$: Velocity vector for fluid dimensional form
${\widetilde{{\varvec{u}}}}_{{\varvec{s}}}=[{\widetilde{u}}_{s},\boldsymbol{ }{\widetilde{v}}_{s}]$: Velocity vector for solid dimensional form
${\varvec{u}}=[u,v]$: Velocity vector for fluids non dimensional form
${{\varvec{u}}}_{{\varvec{s}}}=[{u}_{s},\boldsymbol{ }{v}_{s}]$: Velocity vector for solids non dimensional form
${\varvec{\gamma}}$: Shear strain rate
$$h$$: Artery diameter
$$Ha$$: Hartmann number
$$Re$$: Reynolds number
$$Bn$$: Bingham number
n: Number of iterations
W: Width of the elastic walls
$\zeta$: General solution component
S: PiolaKirchoff stress tensor
$$C$$: Elasticity tensor
ALE: Arbitrary Lagrangian–Eulerian
FSI: Fluid–structure interaction
FEM: Finite element method
MHD: Magnetohydrodynamics
